# The First 6 Years’ Experiences of a National Centralized Offspring Surveillance Setting for Dutch Children Prenatally Exposed to Maternal Cancer to Inform Future International Practice: Protocol for a Demographic Review of Referred Families and Key Lessons Learned

**DOI:** 10.2196/71612

**Published:** 2025-06-24

**Authors:** Evangeline A Huis in 't Veld, Anouk M Kruse, Emma J Verwaaijen, Sterre C Huizer, Elisabeth M van Dijk-Lokkart, Christianne Lok, Maaike Kuethe, Frederic Amant, Mathilde M A van Gerwen, Martine van Grotel, Marry M van den Heuvel-Eibrink

**Affiliations:** 1 Center for Gynecological Oncology Netherlands Cancer Institute Amsterdam The Netherlands; 2 Princess Máxima Center Utrecht The Netherlands; 3 Research Program Cancer Center Amsterdam Amsterdam The Netherlands; 4 Department of Child & Adolescent Psychiatry and Psychosocial Care Amsterdam UMC University of Amsterdam Amsterdam The Netherlands; 5 Child Development Amsterdam Reproduction and Development Amsterdam The Netherlands; 6 Stichting Ster(k) Den Haag The Netherlands; 7 Department of Oncology Unit of Gynaecological Oncology KU Leuven Leuven Belgium; 8 Department of Obstetrics and Gynaecology Division of Gynaecological Oncology UZ Leuven Leuven Belgium

**Keywords:** surveillance, offspring, outpatient clinic, cancer, treatment, exposure in utero, toxic effects

## Abstract

**Background:**

Cancer during pregnancy is a rare and significant life-changing event affecting approximately 1 in 1000-2000 pregnancies. With increasing maternal age and broader application of prenatal screening programs such as the Dutch Non-Invasive Prenatal Testing, incidental detection of maternal cancer is becoming more frequent. Advancements in safe treatment options during pregnancy, supported by the International Network on Cancer Infertility and Pregnancy (INCIP), have led to fewer pregnancy terminations. Consequently, more children are exposed to chemotherapy and other cancer treatments in utero. While short-term safety has been demonstrated for many oncological agents, long-term side effects including physical, neuromotor, neurocognitive, and psychosocial impacts on offspring and their families after delivery are still being assessed. Standard settings for surveillance and care of offspring and their families have, however, never been described.

**Objective:**

Given the importance of expertise in assessing the long-term outcomes of children, the Netherlands established the national centralized Cancer in Pregnancy (CIP) offspring outpatient clinic in 2018, which functions as a standard-of-care surveillance clinic and contributes data to the INCIP registry. Here we provide a demographic overview of referred families and to share (logistic) experiences with the national, centralized, multidisciplinary, and standardized long-term surveillance program for all Dutch children with in utero exposure to maternal cancer and its treatment.

**Methods:**

The CIP offspring outpatient clinic is located at the Princess Máxima Center for Pediatric Oncology and provides surveillance from infancy until 18 years of age. The, relatively small dedicated team, comprising pediatric oncologists, physiotherapists, and a psychological expert, offers a 1-day, multidisciplinary assessment, including physical examinations, neuromotor tests, cardiac monitoring (for anthracycline exposure), renal and auditory screening (for platinum agents), neurocognitive testing, and psychosocial evaluation. Surveillance is aligned with international INCIP guidelines.

**Results:**

From May 2018 to 2024, a total of 226 children (from 221 mothers) have been referred to the CIP offspring outpatient clinic, with 465 follow-up visits completed. The most common maternal cancer types were breast, gynecological, and hematological malignancies. Most women (58%) received chemotherapy during pregnancy; 11% of them had surgery only, 3% underwent radiotherapy, 3% underwent immunotherapy, 16% received a combination of treatment modalities, and 8% did not undergo treatment during pregnancy. Anthracyclines were the most commonly used agents. Median gestational age at delivery was 37.3 weeks. Fourteen percent of the mothers died shortly after delivery, underscoring the emotional and logistical challenges for families.

**Conclusions:**

The CIP offspring outpatient clinic provides a unique, structured approach to long-term surveillance for in utero–exposed children, which enables early detection of potential late effects and provides comprehensive family support. By sharing knowledge and experiences from the unique setting of this national centralized CIP offspring outpatient clinic, this initiative may inspire other countries in developing similar translational facilities to support affected families and improve care worldwide.

**International Registered Report Identifier (IRRID):**

DERR1-10.2196/71612

## Introduction

Cancer during pregnancy is rare, occurring in 1 in 1000-2000 pregnant women [1]. However, this incidence is rising as women tend to delay childbearing [2]. Also, since the introduction of the Non-invasive Prenatal Testing program in the Netherlands (in 2018), maternal cancer, often asymptomatic, is detected incidentally, even before clinical symptoms appear [3]. Hence, cancer during pregnancy is an emerging health and societal challenge, which necessitates dedicated management for the mother and child.

Several decades ago, termination of pregnancy was the standard recommendation for pregnant women with cancer. Today, however, treatment during pregnancy is often feasible and safe. The major shift in this clinical approach is based on evidence from epidemiological studies following children exposed to cancer treatment in utero, primarily led by the International Network on Cancer, Infertility, and Pregnancy (INCIP), founded in 2005 in Europe. This robust framework, now extended to 26 counties worldwide, has significantly expanded our understanding of the long-term effects of in utero exposure to chemotherapy [4-8]. While many chemotherapeutic agents are now considered safe during pregnancy, concerns remain regarding potential long-term toxicities [9-13]. Certain agents, such as anthracyclines and cisplatin, are known to cause long-term cardiotoxicity, nephrotoxicity, and ototoxicity in childhood cancer survivors [9,12,14-17]. Nevertheless, early data suggest that cisplatin may affect hearing, warranting long-term follow-up [2,8,9]. Age-specific cumulative dose limits exist, based mainly on epidemiological data, and placental transfer varies by drug [18,19]. Crucially, prematurity, and not the maternal treatment itself, has been identified as the most significant independent factor that determines affected neurocognitive and motor development in exposed offspring, but the impact of prematurity is similar to that in the healthy population [20]. Preterm birth is known to increase the risk of motor deficits such as coordination difficulties, impaired balance, and fine motor challenges, even in the absence of cerebral palsy [20,21]. Given these risks, prolonging pregnancy whenever feasible is prioritized, with additional chemotherapy courses preferred over premature delivery [2]. These data enhance our understanding and help future expecting patients with cancer and their partners to make informed decisions about the pregnancy complicated by cancer. In most countries, the available program for offspring surveillance is generally dispersed among various and often small centers. Consequently, expertise in the specific field of toxicity in the offspring after exposure to cytotoxic agents is limited.

Since 2018, Dutch pediatric cancer care has been centralized at the Princess Máxima Center, optimizing treatment outcomes and reducing toxicity [22]. Building on this model, the national Cancer in Pregnancy (CIP) offspring outpatient clinic was allowed to be hosted in this center to monitor all Dutch children prenatally exposed to maternal cancer and its treatment. The clinic offers standardized care and long-term surveillance across physical, neuromotor, neurocognitive, and psychosocial domains, integrates research on prenatal treatment effects, and serves as a knowledge hub for health care professionals. Such a centralized setting is currently not available in other countries.

In this overview, we aim to present the design of our unique clinical framework and our experiences from the first 6 years of the CIP offspring outpatient clinic. By sharing our experiences, we aim to encourage the development of similar centralized translational follow-up programs worldwide, improving care by centralization for affected families now and in the future.

## Methods

### Recruitment of Offspring

**Figure 1:**
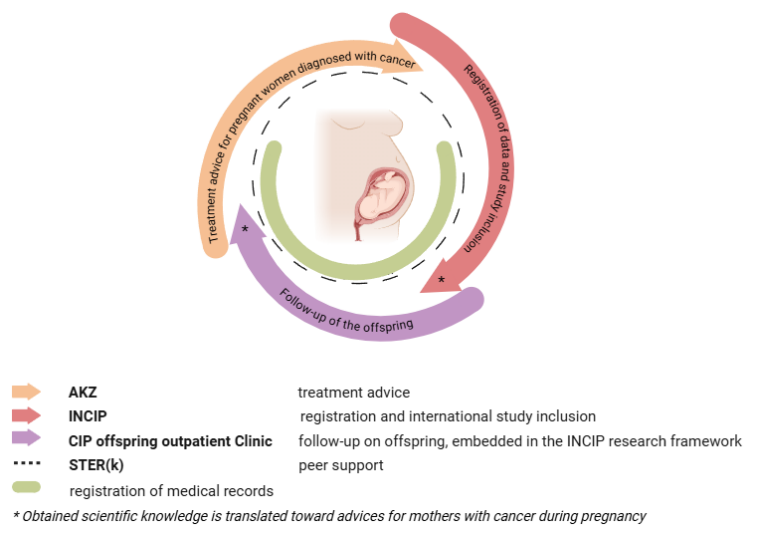
Translational continuum of AKZ, INCIP, and the CIP offspring outpatient clinic. The image was created using BioRender. AKZ: Adviesgroep Kanker en Zwangerschap; CIP: Cancer in Pregnancy; INCIP: International Network on Cancer Infertility and Pregnancy.

In 2012, the Cancer in Pregnancy advisory group (“Adviesgroep Kanker en Zwangerschap”) had been established by Dutch members of the INCIP group [23]. This advisory board (Adviesgroep Kanker en Zwangerschap) has expanded its activities on an international scale now and has evolved into an international participation from countries involved in the INCIP, operating under the name ABCIP (Advisory Board on Cancer, Infertility, and Pregnancy [24]). ABCIP provides multidisciplinary, individualized expert advice for every pregnant patient with cancer within a few days based on evidence from the current literature [23]. This advice is then delivered to physicians responsible for these pregnant patients with cancer. The advisory group comprises a national multidisciplinary team, including oncologists, hematologists, surgeons, radiotherapists, gynecologists, obstetricians, clinical pharmacologists, and scientific researchers, and includes the pediatric oncologists and psychologists responsible for the care of children followed at the CIP offspring outpatient clinic at the Princess Máxima Center in the Netherlands. Referral of a neonate to the national CIP offspring outpatient clinic for standard-of-care follow-up at the Princess Máxima Center is always part of standard advice (Figure 1). This proactive approach identifies potential offspring already at risk in utero.

### Design of the CIP Offspring Care Setting

The surveillance of offspring and their families is conducted at the long-term follow-up outpatient clinic at the Princess Máxima Center, which had been specifically established for childhood cancer survivors in 2018. This area in the hospital, uniquely suited for families exposed to cancer in utero, is separated from the area of direct patient care, thereby limiting the interaction with actively treated children with cancer. We designed this outpatient clinic with all necessary diagnostic facilities (including cardiac ultrasound, electrocardiograms, audiological testing, laboratory facility, pulmonary function testing, neuropsychological and neuromotor testing, as well as clinical consultations) within this designated area, in order to enable the performance of all tests, in 1 day, for the families' convenience, merely, on one and the same location (Multimedia Appendix 1).

The CIP offspring outpatient clinic’s small multidisciplinary team (2 pediatric oncologists specialized in toxicity during and after cancer treatment, 2 physiotherapists, and a psychologist) aims to detect early deviations in physical, neuromotor, neurocognitive, and psychosocial development.

### Standard of Care and Surveillance Strategy

As part of standard care, families are invited for follow-up visits at fixed intervals per the INCIP protocol. Each visit includes a physical and neurological consultation by pediatric oncologists, a neuromotor assessment by a physiotherapist, and limited blood tests for children exposed to chemotherapy in utero (with consent). Cardiac screening is added in case of anthracycline exposure; and nephrological and audiological assessment, after platinum-based exposure. Offspring of mothers with metastatic disease, especially those with histologically confirmed placental involvement, undergo abdominal ultrasounds in the first year to exclude spread of tumor cells to the liver. Identified medical or psychosocial concerns lead to referrals to appropriate specialists, including clinical geneticists, if applicable.

Beyond the context of routine surveillance, for research purposes, internationally standardized, age-appropriate neuropsychological testing is conducted longitudinally, starting from 18 months of age and continuing thereafter [6,7]. Additionally, prior to testing, parents are invited to complete questionnaires regarding general health as well as the executive and behavioral functioning of their child, as part of the neuropsychological investigation [25,26]. All tests are pursued with informed consent. As embedded in the international INCIP research framework, upon obtaining informed consent and anonymization, all collected care data are obtained during regular follow-up care and can be used for research purposes (NCT00330447, METC NL43546.078.13) based on (parental) written informed consent from all participants. The full study protocol is available on the web [27].

Two weeks after the consultations, the results of all tests are shared with the families by telephone. Referral advice, including recommendations for interventions and support in schools, childcare facilities, or educational institutions, can be provided. For this purpose, all results of neuropsychological testing are sent to families by mail. Obviously, we closely communicate with the general practitioner of the families. If any medical, neurocognitive, or developmental concerns arise during clinical care evaluation, children and/or parents are offered to be referred to relevant expert physicians, psychologists, or pediatric physiotherapists, preferably in the region where the families reside, if available. In our experience, this is especially important in families where the mother died after delivery.

### Ethical Considerations

The ethical committee of the Erasmus Medical Center Rotterdam, the Netherlands (METC NL43546.078.13) approved of the study and written (parental) informed consent was obtained for all participants. The study was performed in accordance with the tenets of the Declaration of Helsinki. The full study protocol is available on the web [27], and the study is registered in ClinicalTrials.gov (NCT00330447; first registered on May 26, 2006).

## Results

In this section, we describe the experiences of the CIP offspring outpatient clinic in the first 6 years.

### Epidemiology

**Figure 2:**
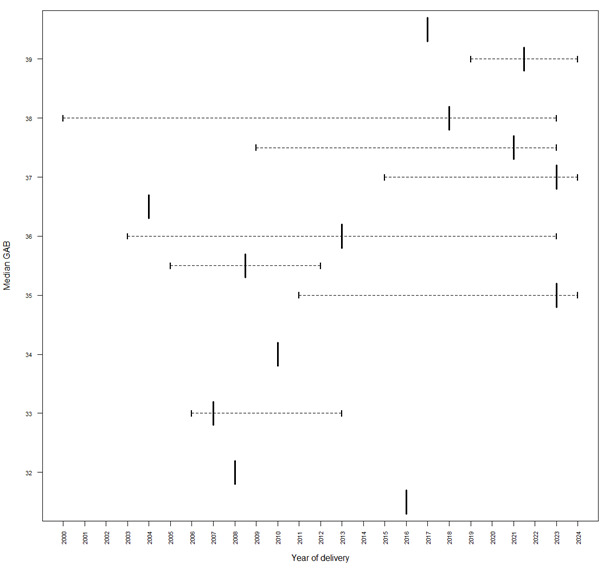
Median gestational age at birth (GAB) of the offspring (n=226).

Since the initial registration of pregnant women with cancer in the INCIP in 2005, a total of 3630 women have been registered, all of whom have given live birth. Of these women, 436 were registered in the Netherlands. Since the centralization of outpatient care for CIP participants’ families in the Netherlands in 2018, a total of 226 children (221 mothers; 5 twins) had been referred to our CIP offspring outpatient clinic (Multimedia Appendix 2). This had already resulted in a combined total of 465 follow-up visits until May 1, 2024 (Figure 2). Of these children, 125 out of 226 (55.33%) were female and 101 (44.7%) were male. The median gestational age at birth was 37.3 (IQR 26.3-41.7) weeks. The distribution of maternal cancers was similar to that in the general population [28]. Disease- and treatment-related characteristics are presented in Table 1. Moreover, we observed an increase in gestational age at birth over time, which is likely attributable in part to the implementation of evidence-based recommendations by the multidisciplinary advisory board aimed at minimizing preterm delivery.

As of May 2024, thirty out of 221 (14%) mothers had died due to cancer early after delivery. Twelve of these mothers died of breast cancer, 5 died of a hematological malignancy, 4 died of gastrointestinal cancer, 3 died of cervical cancer, 3 died of melanoma, 2 died of bone cancer, and 1 died of a rhabdomyosarcoma. Overall, the children appear to be doing well medically. However, of the 226 children, we did observe 1 case of a neuroblastoma (stage IV) in a child at the age of 3 years and treatment was administered. This boy had been born from the mother with a meningioma and had been exposed to surgery and anesthesia in utero (at gestational age 22 weeks). This child is under regular cancer follow-up care after his cancer diagnosis at the Princess Máxima Center now.

We recognized that being diagnosed with cancer during pregnancy is a significant life event for the entire family, which can evoke symptoms of psychological concern. We noticed that the medical care and psychosocial support of the small dedicated core team, the focus on the long-term care plan and surveillance, and attention to the entire family system are highly appreciated by the families in general. Parents particularly appreciate being reassured, especially in the early stages following a stressful pregnancy, that their child is progressing along expected developmental lines. They value being well-informed about their child’s development, and the combination of a small, dedicated team and the logistical framework we have developed for our clinic proves to be highly effective in supporting the parents.

**Table 1:** Disease and treatment-related characteristics of the mothers.

Characteristic		Children, n (%)
**Maternal cancer diagnosed during pregnancy (n=226; 5 twins)**		
	Breast	108 (47.78)
	Gynecological	40 (17.96)
	Hematological	30 (13.27)
	Melanoma	21 (9.29)
	Gastrointestinal	7 (3.09)
	Brain tumor	4 (1.70)^a^
	Bone	3 (1.33)
	Urothelial	3 (1.33)
	Other	10 (4.42)
**Chemotherapeutic agent type (n=132)**		
	Anthracyclines	109 (48.23)
	Taxanes	65 (28.76)
	Platinum	40 (17.70)
	Other	12 (5.31)
**Timing radiotherapy (at gestational age in weeks; n=7)**		
	7-13	1 (14.28)
	11-14	1 (14.28)
	18-23	1 (14.28)
	20-27	1 (14.28)
	27-34	1 (14.28)
	Unknown	2 (28.57)
**Immunotherapy (targeted) type (n=6)**		
	PEGylated interferon	1 (16.67)
	Nilotinib	1 (16.67)
	Bosutinib	1 (16.67)
	Trastuzumab	1 (16.67)
	Trastuzumab + pertuzumab	1 (16.67)
	Interferon + nilotinib	1 (16.67)

^a^One (25%) later turned out to be a meningioma.

### Peer Support

During the initial years of the CIP offspring outpatient clinic, parents took the initiative to establish a peer support Association—Stichting STER(k). This association provides support to parents and partners through information, a website, and on-demand support activities [29]. Stichting STER(k) provides peer support for women, their partners, and families facing cancer during pregnancy and in the postpartum period, thereby addressing intensive treatment for the mother and the needs of the newborn, which often create logistical challenges. The association strongly collaborates with our CIP offspring outpatient clinic team to disseminate general information by covering topics such as psychological and social issues and logistic aspects of a pregnancy complicated by a cancer (treatment) and practical issues such as breastfeeding, maternity care, and maternal health. Organization of parent-information workshops has been initiated.

## Discussion

This paper outlines the mission, design, and logistics of the Dutch CIP offspring outpatient clinic, the first nationally centralized clinic for children exposed to cancer in pregnancy, and shares key lessons learned.

### Experiences of the CIP Offspring Team

We consider this teamwork, the establishment of Stichting STER(k), and the continuum with the ABCIP and INCIP groups, which identify the children already during pregnancy, as well as the translational set-up, that is, the contribution of the outcome data to the INCIP registry from the CIP offspring outpatient clinic (and the studies carried out in other countries) to provide knowledge that informs the ABCIP [24], as keys to success.

We have learned that gestational age is the most significant independent predictive factor for long-term neurocognitive impairment rather than in utero exposure to cancer treatment. As we currently discourage premature delivery, whenever feasible over time, we observed that prematurity consequently appears to decrease over time. Furthermore, we have observed inconsistent national referrals due to lack of expertise. This requires ongoing effort.

Finally, our nationally centralized structure is unique within the INCIP research framework, as follow-up in other countries is typically decentralized or is currently not taking place. Sharing our experiences as well as international communication may support similar structured follow-up in other countries, ultimately improving global outcomes for children prenatally exposed to maternal cancer.

### Limitations

Long-term effects of maternal cancer treatment during pregnancy, especially beyond adolescence, remain largely unknown due to limited very-long-term follow-up data (>10 years). This is crucial, as late effects, such as anthracycline-induced cardiotoxicity, may emerge decades later, similar to what has been observed in children with cancer [30-37]. Ongoing surveillance through adolescence and adulthood is essential to uncover potential risks in physical, cognitive, emotional, and psychosocial development. Collaboration with the INCIP research framework and the advisory board has provided valuable insights, suggesting that early care may be more targeted, focusing on specific evidence-based risk factors, and shifting the emphasis toward the very-long-term effects of prenatal exposure to maternal cancer treatment.

Furthermore, most knowledge about long-term side effects for offspring is available from large cohort studies with childhood cancer survivor with conventional chemotherapeutic agents. The rapid development of novel innovative cancer treatments poses the need for additional risk exploration, as some of them had not been used in children so far [38-41]; hence, in vitro exploration is required before these treatments can be safely applied in pregnant women with cancer [42].

In pediatric oncology, where the median age of the first childhood cancer survivors is now approaching 40 years, the long-term effects of chemotherapy exposure are gradually becoming clear. The CIP offspring outpatient clinic, with shorter follow-up so far, highlights the need for lifelong, individualized monitoring similar to that in childhood cancer survivors. Families often need additional support to cope with the complex postpartum period; referrals are made to specialized care facilities and peer support organizations like Stichting STER(k), in collaboration with general practitioners. To improve care, our goal is full cohort inclusion through increased awareness and standardized, risk-adaptive screening that is timed appropriately for different toxicities. Lifelong multidisciplinary follow-up and further research, including molecular studies, are vital to fully understand and support this unique population.

### Conclusions

In conclusion, we have found that the establishment of a dedicated national centralized CIP offspring outpatient clinic for exposed offspring of pregnant women with cancer during their pregnancy who are receiving treatment addresses a clear need for structured care and support for families, while providing standardized long-term surveillance and consistent data collection on the health and development of exposed children. Given the relative rarity of this condition, this setup in the Netherlands, which provides this care in a nationwide expert setting with the aim of providing optimal care for children and their families, may serve as a model for other countries that aim to establish a similar national centralized program. This paper may help enhance connections with other countries that envisage building a similar structure.

## Appendix

Multimedia Appendix 1 Design of the LATER-outpatient clinic embedded in the Princess Máxima Center.

Multimedia Appendix 2 Recruitment.

## Abbreviations

ABCIP: Advisory Board on Cancer, Infertility, and Pregnancy
CIP: Cancer in Pregnancy
INCIP: International Network on Cancer Infertility and Pregnancy

## Data Availability

All data generated or analyzed during this study are included in this published article.
