# Supplementary figures and images for: The First 6 Years’ Experiences of a National Centralized Offspring Surveillance Setting for Dutch Children Prenatally Exposed to Maternal Cancer to Inform Future International Practice: Protocol for a Demographic Review of Referred Families and Key Lessons Learned
Source: JMIR Res Protoc. 2025 Jun 24;14:e71612. doi: 10.2196/71612 (PMC12238779; doi:10.2196/71612)

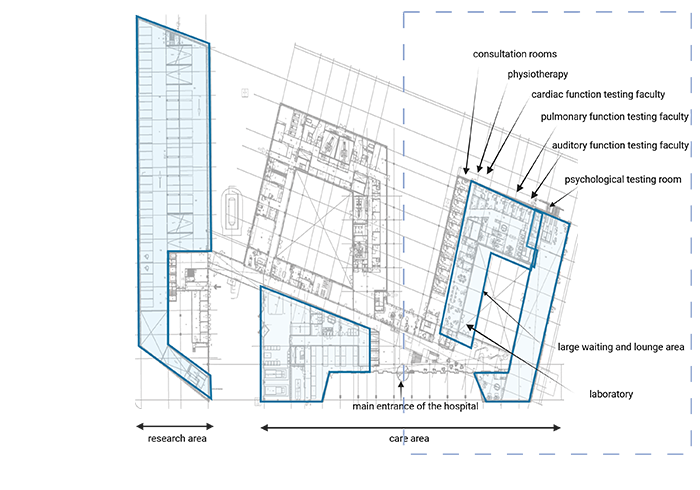

Supplement: Multimedia Appendix 1 [file resprot_v14i1e71612_app1.png]

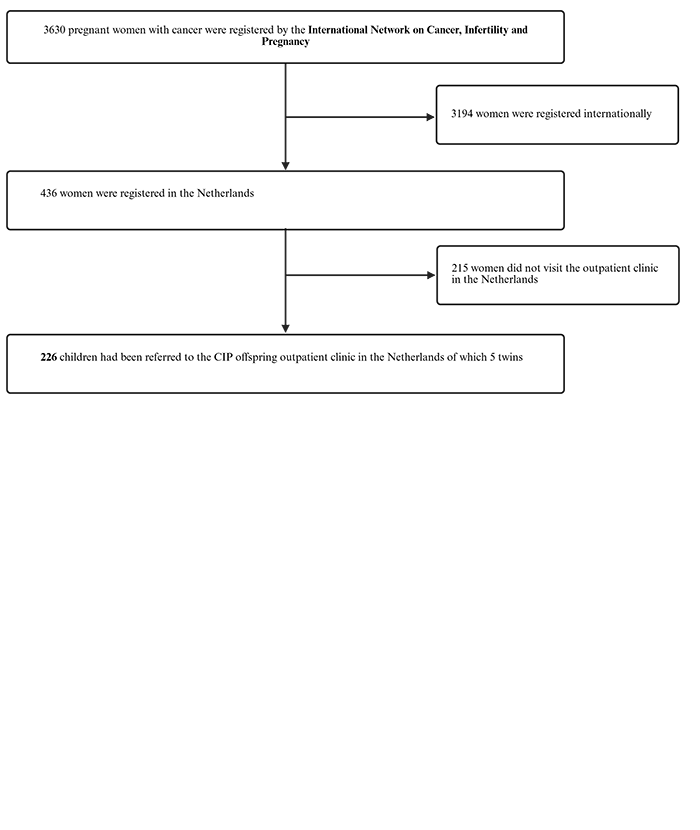

Supplement: Multimedia Appendix 2 [file resprot_v14i1e71612_app2.png]
